# Inhibiting De Novo Biosynthesis of Ceramide by L-Cycloserine Can Prevent Light-Induced Retinal Degeneration in Albino BALB/c Mice

**DOI:** 10.3390/ijms252413389

**Published:** 2024-12-13

**Authors:** Faiza Tahia, Dejian Ma, Daniel J. Stephenson, Sandip K. Basu, Nobel A. Del Mar, Nataliya Lenchik, Harry Kochat, Kennard Brown, Charles E. Chalfant, Nawajes Mandal

**Affiliations:** 1 Department of Pharmaceutical Sciences, University of Tennessee Health Science Center, Memphis, TN 38163, USA; ftahia@uthsc.edu (F.T.); dma6@uthsc.edu (D.M.); 2 Department of Ophthalmology, University of Tennessee Health Science Center, Memphis, TN 38163, USA; sbasu8@uthsc.edu (S.K.B.); ndelmar@uthsc.edu (N.A.D.M.); nlenchik@uthsc.edu (N.L.); 3 Departments of Medicine and Cell Biology, University of Virginia School of Medicine, Charlottesville, VA 22903, USA; stephensondj@alumni.vcu.edu (D.J.S.); krj2sf@uvahealth.org (C.E.C.); 4 Plough Center for Sterile Drug Delivery Solutions, University of Tennessee Health Science Center, Memphis, TN 38163, USA; hkochat@uthsc.edu (H.K.); kbrown@uthsc.edu (K.B.); 5 Research Service, Richmond Veterans Administration Medical Center, Richmond, VA 23298, USA; 6 Department of Anatomy and Neurobiology, University of Tennessee Health Science Center, Memphis, TN 38163, USA; 7 Memphis VA Medical Center, Memphis, TN 38104, USA

**Keywords:** retinal degeneration, photoreceptor cell death, ceramide, L-Cycloserine, light-induced retinal degeneration (LIRD), BALB/c mice, pharmacokinetics

## Abstract

Retinal degenerative diseases lead to irreversible vision loss due to photoreceptor cell death, driven by complex genetic and environmental factors. Ceramide, a sphingolipid metabolite, emerges as a critical mediator in the apoptotic cascade associated with retinal degeneration. Our previous work demonstrated L-Cycloserine’s ability to protect photoreceptor-derived cells from oxidative stress by inhibiting the de novo ceramide pathway and thus prompting further investigation on its effect in the in vivo retina. This study investigates the potential of L-Cycloserine to protect albino BALB/c mice against light-induced retinal degeneration (LIRD). L-Cycloserine, in an optimal dose, administered systemically 30 min before LIRD, was found to prevent photoreceptor cell death significantly from light-induced degeneration. We further determined the retinal bioavailability and pharmacokinetic behavior of L-Cycloserine, its effect on sphingolipid profile, expression of sphingolipid biosynthetic, and cell death-promoting genes and proteins from the retina to understand the underlying mechanisms. This study lays the groundwork for further preclinical and clinical investigations into L-Cycloserine’s potential as a novel therapeutic in treating retinal degenerative diseases.

## 1. Introduction

The hallmark characteristic of major retinal degenerative diseases like Retinitis Pigmentosa (RP) and age-related macular degeneration (AMD) is the apoptotic death of photoreceptor cells, which ultimately leads to irreversible vision loss or blindness, that has a profound impact on a person’s quality of life. The number of people affected by AMD is approximately 196 million, while an estimated 1.5 million individuals are affected by RP in the age group of 20–64 years [1]. Despite the severity and the number of people affected by these diseases, effective therapeutic options are limited due to complex pathophysiology governed by a combination of genetic and environmental factors [2]. The genetic component of retinal degeneration presents a multitude of complexities, as evidenced by the identification of over 250 genes associated with monogenic forms of the disease [3]. These genes encode proteins involved in various structural and functional aspects of photoreceptors, retinal pigment epithelial (RPE) cells, and other retinal neurons [4]. Consequently, targeting a single gene through gene therapy may not sufficiently address the multifaceted nature of the condition. Therefore, interventions targeting second messengers might provide a more comprehensive approach to therapy.

Ceramide, the sphingolipid metabolite, emerges as a critical second messenger implicated in apoptosis, and elevated levels have been linked to cellular toxicity, inflammation, and cell death [5,6,7,8,9]. Although ceramide has essential roles in membrane structure and cell signaling, increased ceramide levels have been associated with photoreceptors and RPE cell death [10,11,12,13]. Studies conducted both in vitro and in vivo have consistently demonstrated a correlation between ceramide elevation and cell death across various retinal degeneration model systems. In vitro studies utilizing human RPE-derived cells [13], rat retinal neuronal cultures [10], and mouse photoreceptor-derived 661 W cells [11,14] have highlighted the association between ceramide accumulation and cell death. In investigations involving in vivo retinal degeneration, ceramide has been identified as the primary factor responsible for inducing apoptosis in photoreceptors, as observed in the rd10 mouse model [15], rabbit retinal detachment model [16], AdipoR1–/– mice [17], Drosophila mutants [18], light-induced degeneration in Sprague Dawley (SD) rats [19] and transgenic Rhodopsin mutant P23H1 rat model [7]. This underscores the prospect of therapeutic advancement in treating retinal degenerative diseases by targeting ceramide. Given its ubiquitous role in the pathophysiology of various retinal degenerative diseases, such an approach holds the potential for a more comprehensive pharmacotherapeutic impact, offering a broadly applicable strategy for managing these conditions.

The onset and progression of retinal degenerative diseases are attributed to several factors, including prolonged exposure to intense white light [20,21,22], oxidative stress caused by free radicals [23,24,25,26], and inflammatory responses [23,27]. Our study explores the potential involvement of ceramide in the process of photoreceptor cell death in a light-induced retinal degeneration (LIRD) model. In LIRD, extended exposure of the retina to light initiates photochemical reactions in both photoreceptors and RPE cells. This process leads to the accumulation of reactive oxygen species (ROS) and the subsequent oxidation of lipids and proteins, features that are characteristic of AMD and other degenerative retinopathies [28,29,30,31,32]. Originating in 1966 through the work of Noell et al. [33], LIRD has been widely utilized as a model, both independently and in conjunction with genetic knockout models, for investigating the mechanisms behind photoreceptor cell death and screening neuroprotective compounds for effectiveness. This model’s controllable light exposure makes it particularly useful, and it replicates apoptotic cell death observed in hereditary retinal degenerations, including conditions such as RP and AMD [32].

Several commercially available ceramide biosynthesis inhibitors, including Myriocin, Fumonisin B1, and Fingolimod (FTY720), have been explored in terms of their effect on retinal degeneration. Advancements in this field include the examination of Myriocin as a ceramide biosynthesis inhibitor to mitigate retinal degeneration in rd10 mice [34]. Additionally, Myriocin administration through various routes in the Tvrm4 mice model of autosomal dominant RP correlated with reduced retinal ceramides and preservation of electroretinogram (ERG) responses [15,35]. Our previous investigations have also showcased the efficacy of systemic administration of FTY720, a ceramide synthase (CerS) inhibitor, in preventing elevated retinal ceramide levels and protecting photoreceptor cells from LIRD in SD rats [19] and in transgenic Rhodopsin mutant P23H1 rat model [7]. 

In this study, we have investigated the protective effect of a small molecule, L-Cycloserine, on photoreceptor cell death in the LIRD model of albino BALB/c mice. Cycloserine is a cyclic analog of serine and/or alanine and has been demonstrated to be an irreversible inhibitor of various pyridoxal 5′-phosphate (PLP)-dependent enzymes, including transaminases, racemases, and decarboxylases [14,36]. The initial and rate-limiting step in de novo ceramide biosynthesis is catalyzed by the PLP-dependent enzyme Serine-palmitoyl transferase (SPT), and L-Cycloserine is reported to be a highly effective SPT inhibitor, exhibiting a 100-fold greater inhibition compared to its enantiomer, D-Cycloserine [37]. Consequently, it is reasonable to anticipate that L-Cycloserine, acting as a potent SPT inhibitor, can decrease ceramide levels and other complex sphingolipids within a cell.

Drawing from the established role of L-Cycloserine as a de novo ceramide biosynthesis inhibitor explored in various investigations [38,39,40], our previous study demonstrated that L-Cycloserine protects mouse photoreceptor-derived 661 W cells from oxidative stress-induced damage by inhibiting de novo ceramide synthesis [14]. Thus, in continuation of our exploration of L-Cycloserine as a promising therapeutic candidate for preventing or delaying the onset or progression of retinal degeneration, this study hypothesizes that L-Cycloserine will protect photoreceptor cell death from LIRD in BALB/c mice by inhibiting the de novo ceramide biosynthesis pathway. Our study further investigates the optimum systemic dose of L-Cycloserine needed for its protective effect on BALB/c mice photoreceptors from LIRD. The bio-distribution of L-Cycloserine and its effect on sphingolipid composition is also analyzed to understand L-Cycloserine-mediated protection in light-stressed BALB/c mice retina. The findings of this study demonstrate that L-Cycloserine significantly protects BALB/c mice retina from light-induced degeneration. It is noteworthy that L-Cycloserine has not been previously explored in any retinal degeneration model, and the outcomes of this study may, therefore, contribute to the successful evaluation of L-Cycloserine as a therapeutic approach for retinal degeneration in preclinical models, thereby laying the foundation for potential future clinical investigations.

## 2. Results

### 2.1. L-Cycloserine Protects BALB/c Mice Retina from Light-Induced Degeneration

**Figure 1:**
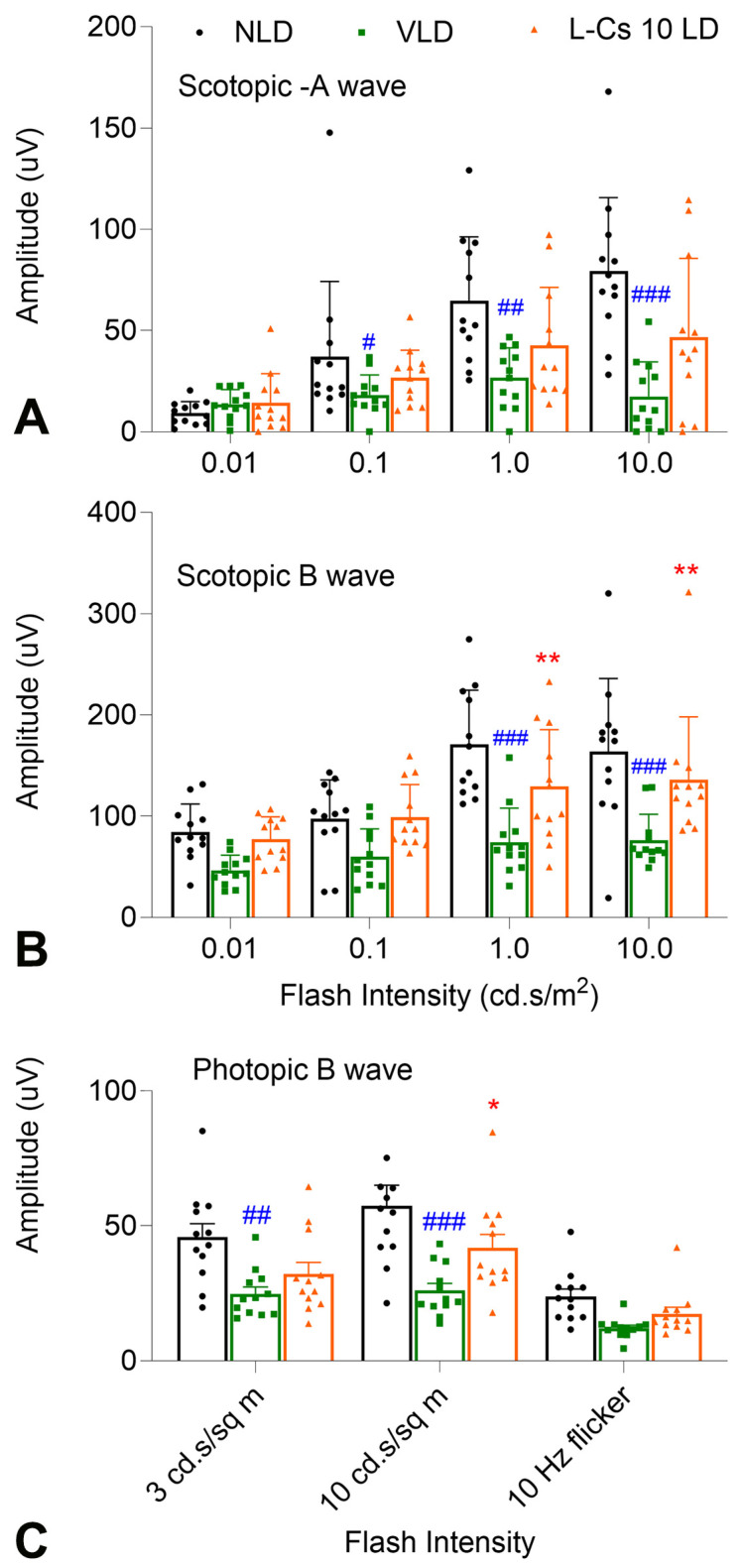
L-Cycloserine protects mouse retina from LIRD. ERG analysis shows Scotopic a-wave (**A**), b-wave (**B**), and Photopic b-wave (**C**) from NLD: no light-damaged control group; VLD: vehicle-injected light-damaged group; L-Cs 10 LD: L-Cycloserine-treated (10 mg/kg) light-damaged group. (n = 6/group; values are mean ± SEM; * represents significance between VLD and L-Cs 10 LD; * *p* < 0.05, ** *p* < 0.01, by the student *t* test; # represents significance between NLD and VLD; # *p* < 0.05, ## *p* < 0.01, ### *p* < 0.001, by the student *t* test).

To test whether L-Cycloserine can protect BALB/c mice retina from light-induced degeneration, a dose of 10 mg/kg of L-Cycloserine was chosen to be administered to BALB/c mice (L-Cs 10 LD) by intraperitoneal (IP) injection at 0.5 h before the start of light exposure. Vehicle (sterile water) injected light-damaged mice (VLD), and no light-damaged mice (NLD) were used as control. Seven days post light damage, ERG data revealed significant retinal damage in VLD mice, evident in the significant reduction of scotopic a and b wave amplitudes, as well as photopic b wave amplitude (Figure 1A, B and C, respectively). Notably, treatment with L-Cycloserine at a dose of 10 mg/kg (L-Cs 10 LD) exhibited significant protection by preserving scotopic and photopic b wave responses (Figure 1B and C, respectively). Additionally, no change in body weight was observed in these mice from the time of treatment to this ERG test.

**Figure 2:**
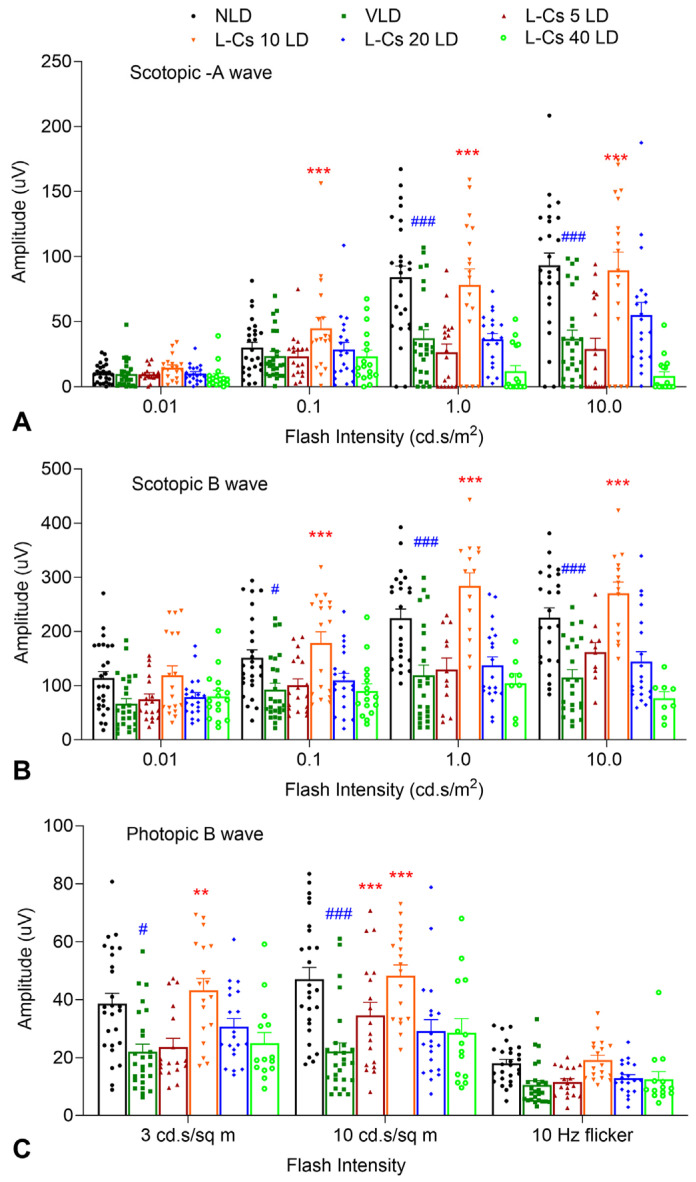
A 10 mg/kg dose of L-Cycloserine provides maximum protection of mouse retina from LIRD. ERG analysis shows Scotopic a-wave (**A**), b-wave (**B**), and Photopic b-wave (**C**) from NLD: no light-damaged control group; VLD: vehicle-injected light-damaged group; L-Cs 5 LD,10 LD, 20 LD, and 40 LD: L-Cycloserine-treated (5, 10, 20, and 40 mg/kg, respectively) light-damaged group. n = 13 (NLD, VLD); n = 9 (L-Cs 5 LD, L-Cs 10 LD); n = 10 (L-Cs 20 LD); n = 8 (L-Cs 40 LD); values are mean ± SEM; * represents significance between VLD and L-Cycloserine treated group; ** *p* < 0.01, *** *p* < 0.001, by the student *t* test; # represents significance between NLD and VLD; # *p* < 0.05, ### *p* < 0.001, by the student *t* test).

To determine the most effective dose of L-Cycloserine, four single doses (5, 10, 20, and 40 mg/kg) were tested using the same protocol as mentioned above. Similar to the previous results, ERG data indicated significant retinal damage in VLD mice, characterized by a significant decrease in scotopic a and b wave amplitudes and photopic b wave amplitude (Figure 2A, B and C, respectively). As observed earlier, L-Cycloserine treatment at a dose of 10 mg/kg (L-Cs 10 LD) provided significant protection by preserving scotopic a and b waves, as well as photopic b wave responses, compared to doses of 5 and 20 mg/kg (L-Cs 5 LD and L-Cs 20 LD), while no protection was observed at 40 mg/kg, L-Cs 40 LD (Figure 2A, B and C, respectively).

**Figure 3:**
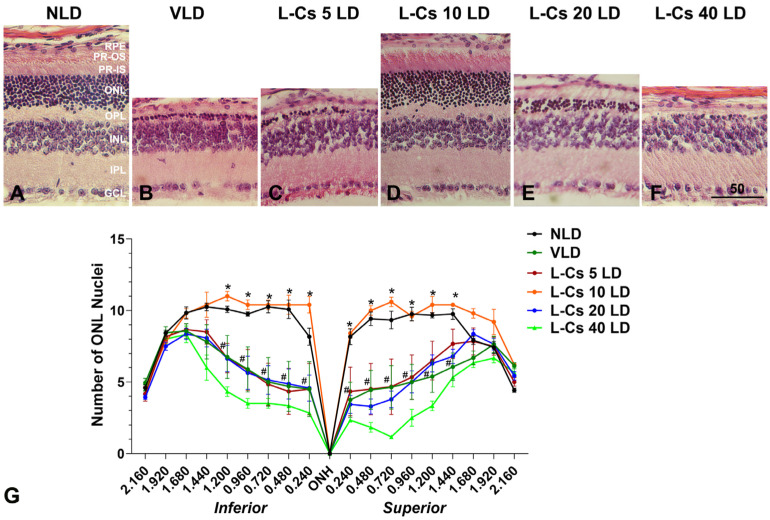
A 10 mg/kg dose of L-Cycloserine provides maximum protection of mouse retina from LIRD. Representative retinal histological sections from each treatment: (**A**): NLD, no light-damaged control; (**B**): VLD, vehicle-injected light-damaged group; (**C**–**F**): L-Cs 5 LD,10 LD, 20 LD, and 40 LD, represents L-Cycloserine-treated (5, 10, 20, and 40 mg/kg, respectively) light-damaged group. Scale bar in F represents 50 microns. (**G**) Quantitative morphometric measurement of ONL nuclei count from H and E-stained slides. n = 12 (NLD); n = 16 (VLD); n = 10 (L-Cs 5 LD); n = 13 (L-Cs 10 LD); n = 14 (L-Cs 20 LD); n = 11 (L-Cs 40 LD). Values are mean ± SEM; * represents significance between VLD and L-Cycloserine treated group; * *p* < 0.001, by the student *t* test; # represents significance between NLD and VLD; # *p* < 0.001, by the student *t* test. Abbreviations for retinal section: RPE, retinal pigment epithelium; PR, photoreceptors; OS, outer segments; IS, inner segments; ONL, outer nuclear layer; OPL, outer plexiform layer; INL, inner nuclear layer; IPL, inner plexiform layer; GCL, ganglion cell layer.

Representative H&E-stained sections of the retina 7 days after light damage were examined (Figure 3A–F). By 7 days, the dead photoreceptors are cleared by macrophages, and the remaining nuclei in the Outer Nuclear Layer (ONL) represent the surviving photoreceptors. The VLD group displayed significant photoreceptor loss (number of nuclei in the ONL), particularly in the central retina (Figure 3B), revealing damage induced by light as expected. Treatment with L-Cycloserine at a dose of 10 mg/kg (L-Cs 10 LD, Figure 3D) provided significant protection from light-induced photoreceptor cell death since the number of nuclei in the ONL displayed is very close to NLD (Figure 3A). By quantitative morphometry, the number of ONL nuclei in the entire retina was counted from the superior to inferior end through the vertical meridian, and values were plotted as a spider graph (Figure 3G). NLD displays an ONL thickness as expected of mice with normal vision. VLD reveals photoreceptor degeneration (greater than 50% damage) induced by light with a significantly reduced number of ONL nuclei. L-Cs 10 LD demonstrated the maximum preservation of photoreceptors compared to other doses, showing the number of ONL nuclei very similar to NLD and demonstrating a significant difference from the VLD group (Figure 3G). Doses of 5 and 20 mg/kg (L-Cs 5 LD and L-Cs 20 LD) showed no significant difference in number of ONL nuclei compared to VLD, while the 40 mg/kg dose (L-Cs 40 LD) failed to protect photoreceptors from light-induced degeneration, exhibiting a significantly reduced number of ONL nuclei compared to VLD (Figure 3G).

In summary, these findings indicate that L-Cycloserine can effectively protect the BALB/c mice retina from light-induced degeneration when administered in an optimum dose, with the 10 mg/kg dose demonstrating maximum protective effect.

### 2.2. L-Cycloserine Up to a Dose of 40 mg/kg Shows No Toxicity in BALB/c Mice Retina

The dose efficacy assessment of L-Cycloserine in light-induced degenerated BALB/c mice retina using ERG indicates that 40 mg/kg dose of L-Cycloserine (L-Cs 40 LD) failed to protect the BALB/c mice retina from light-induced damage (Figure 2A–C). Histopathological analysis further reveals a significant loss of the ONL nuclei corresponding to L-Cs 40 LD compared to VLD (Figure 3F,G). Consequently, it is crucial to discern whether this toxic effect of a high dose of L-Cycloserine in light-induced degenerated BALB/c mice retina is solely a drug-induced effect or a synergistic consequence of light exposure and the presence of a high dose of ceramide inhibitor, L-Cycloserine. Thus, retinal function and morphology are evaluated in this study through ERG and histology seven days after L-Cycloserine administration to healthy BALB/c mice at its most protective (10 mg/kg, L-Cs 10) and least protective (40 mg/kg, L-Cs 40) doses, without any light exposure.

ERG data reveal no significant difference in retinal function between mice injected with sterile water (Vehicle) and those injected with L-Cycloserine at doses of 10 and 40 mg/kg (L-Cs 10 and L-Cs 40, respectively) (Supplementary Figure S1A–C). All scotopic and photopic ERG responses between the two doses, L-Cs 10 and L-Cs 40, also show no significant difference (Supplementary Figure S1A–C). Histopathological results, illustrated through representative H and E-stained sections and a spider diagram, also demonstrate no significant difference in number of ONL nuclei between vehicle and L-Cycloserine injected groups: L-Cs 10 and L-Cs 40 (Supplementary Figure S2A–D). In summary, these findings suggest that L-Cycloserine, up to a dose of 40 mg/kg, does not have any detrimental effect on BALB/c mice’s retinal function and morphology when evaluated after seven days. The observed effect of L-Cycloserine at a dose of 40 mg/kg, when injected 0.5 h before light damage, may thus be a synergistic outcome of light exposure or stress, together with the presence of a sphingolipid inhibitor, which needs further exploration.

### 2.3. L-Cycloserine Distributes to Various Tissues and Reaches the Target Tissue—The Retina

**Figure 4:**
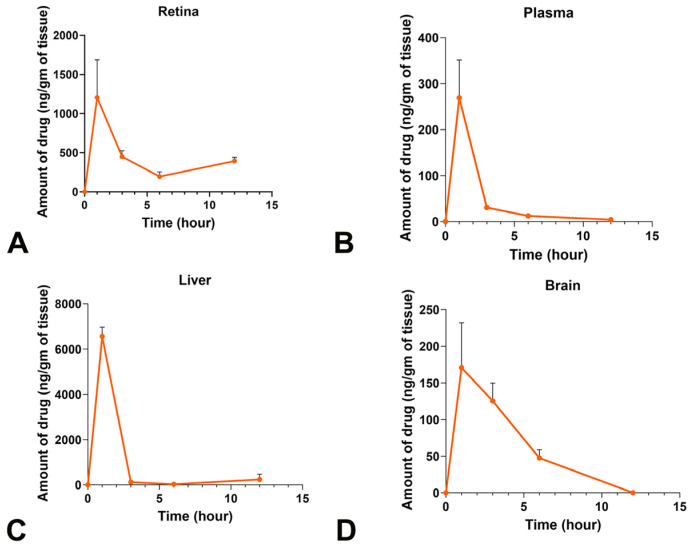
Pharmacokinetic profile of L-Cycloserine in plasma and various tissues. L-cycloserine was intraperitoneally injected at a dose of 20 mg/kg and quantified using LC-MS/MS in retina (**A**), plasma (**B**), liver (**C**), and brain (**D**) at various time points after administration. The results are expressed as a mean ± SEM (n = 7 for each time point).

To comprehend the retinal protective effect of L-Cycloserine from light damage, it is crucial to assess whether the drug effectively reaches the target tissue, the retina and also understand its overall pharmacokinetic behavior. Therefore, L-Cycloserine was administered via the IP route at a dose of 20 mg/kg to healthy BALB/c mice, and the drug concentration was quantified using LC-MS/MS in various tissues, including the retina, liver, brain, and plasma at predetermined time points (Figure 4), to assess the biodistribution of the drug. The results demonstrate that L-Cycloserine is absorbed into the plasma, distributed to the liver and the target tissue of the retina, and passes the blood–brain barrier to reach the brain (Figure 4A–D). The peak concentration of the drug is uniformly achieved across all investigated tissues around 1–2 h post-injection, and the majority of the drug is eliminated within 3 h (Figure 4A–D).

### 2.4. Light Stress Alters Specific Sphingolipid Species in BALB/c Retina

**Figure 5:**
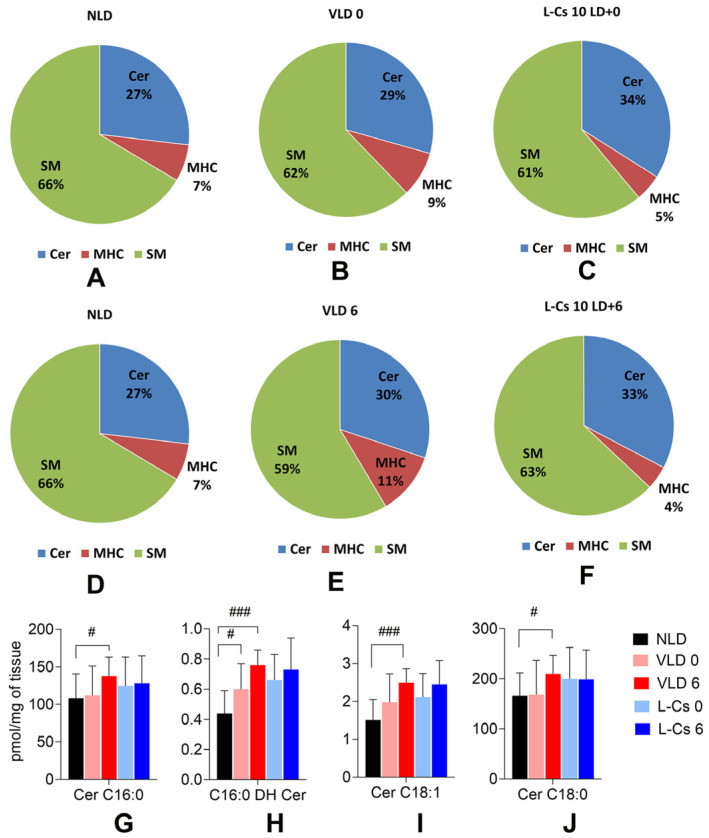
Light damage and L-Cycloserine treatment alter ceramide and other sphingolipid levels in light-damaged BALB/c mice retina. (**A**–**F**): Analysis of major sphingolipid levels by absolute value (pmol/mg of tissue) showing the total composition of ceramide (Cer), sphingomyelin (SM), and monohexosylceramide (MHC) percent in BALB/c mice retina (n = 10/group). Analysis of selected ceramide species by absolute value (pmol/mg of tissue) in BALB/c mice retina at 0 h and 6 h after light damage (**G**–**J**). (n = 10/group, values are mean ± SEM; # represents significance between NLD and VLD; # *p* < 0.05, ### *p* < 0.001, by student *t*-test). NLD: no light-damaged control group, VLD 0: vehicle-injected light-damaged group at 0 h after light damage, L-Cs 0: L-Cycloserine (10 mg/kg) treated light-damaged group at 0 h after light damage, VLD 6: vehicle-injected light-damaged group at 6 h after light damage and L-Cs 6: L-Cycloserine (10 mg/kg) treated light-damaged group at 6 h after light damage.

To investigate the impact of light stress and L-Cycloserine treatment on sphingolipids in BALB/c mice retinas, ceramide (Cer), monohexosylceramide (MHC), and sphingomyelin (SM) species were analyzed immediately (0 h) and 6 h after light damage in all three groups of mice: no light-damaged control group (NLD); vehicle-injected, light-damaged group (VLD); and L-Cycloserine-injected (L-Cs 10 LD) light-damaged group. Analysis of the relative percentages or fractions of Cer, MHC, and SM at LD + 0 and LD + 6 h revealed distinct patterns. Immediately following light damage (LD + 0), the sum of the percentage of Cer and MHC increased slightly from 34% (NLD: Cer 27% + MHC 7%) to 38% (VLD: Cer 29% + MHC 9%) (Figure 5A,B). However, with the treatment of L-Cycloserine, the percentage stayed back to 39% (L-Cs 10 LD: Cer 34% + MHC 5%) (Figure 5C). The percentage of SM decreased from 66% to 62% upon light exposure, while treatment with L-Cycloserine kept it at 61% (Figure 5A–C). Six hours after light damage (LD + 6), the sum of the percentage of Cer and MHC increased from 34% (NLD: Cer 27% + MHC 7%) to 41% (VLD: Cer 30% + MHC 11%) (Figure 5D,E). However, when treating L-Cycloserine, the percentage reverted to 37% (L-Cs 10 LD: Cer 33% + MHC 4%) (Figure 5F). The percentage of SM decreased from 66% to 59% upon light exposure, while treatment with L-Cycloserine brought it back to 63% (Figure 5D–F).

Different species of ceramide and its metabolites play diverse roles in cellular processes, including stress response [41]; thus, individual ceramide species in retinal tissue were also evaluated by their absolute values (pmol/mg of tissue), 0 and 6 h after light damage (Figure 5G–J). Immediately following light damage (LD + 0), there was a significant increase in dihydroceramide species C16:0 DH (*p* < 0.05, Figure 5H), whereas 6 h after light damage, there was a significant increase in ceramide species C16:0 (*p* < 0.05, Figure 5G), C16:0 DH (*p* < 0.001, Figure 5H), C18:1 (*p* < 0.001, Figure 5I) and C18:0 (*p* < 0.05, Figure 5J), when compared to NLD. Treatment with L-Cycloserine (L-Cs 10 LD) reduced all these species from their heightened levels.

### 2.5. L-Cycloserine Modulates Expression of Specific Genes in Light-Damaged BALB/c Mice Retina

**Figure 6:**
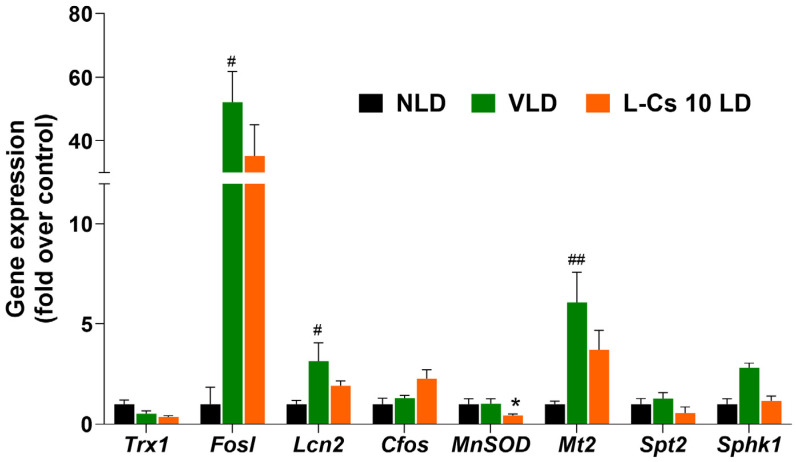
L-Cycloserine modulates retinal gene expression in light-damaged BALB/c mice. Quantitative analysis of gene expression of no light-damaged control (NLD), vehicle-injected light-damaged (VLD), and L-Cycloserine (10 mg/kg) treated light-damaged (L-Cs 10 LD) retina 0 h after light damage. Expression values (±SEM) are presented against fold change over control value (NLD), which was set to 1.0 (n = 6; # represents significance between NLD and VLD; # *p* < 0.05, ## *p* < 0.01, by the student *t* test; * represents significance between VLD and L-Cs 10 LD; * *p* < 0.05, by the student *t* test).

The expression of a series of genes related to the antioxidant, apoptotic, and sphingolipid pathways was investigated immediately after light damage in all three groups of mice: control no light-damaged (NLD); vehicle-injected light-damaged (VLD); and L-Cycloserine-injected (L-Cs 10 LD) light-damaged group, using qRT–PCR (Figure 6). Compared to control NLD, the light-damaged group (VLD) exhibited a significant alteration in the expression of *Fosl* (Figure 6, 52.17-fold increase, *p* < 0.05), *Lcn2* (Figure 6, 3.15-fold increase, *p* < 0.05) and *Mt2* (Figure 6, 6.08-fold increase, *p* < 0.01). Treatment with L-Cycloserine (L-Cs 10 LD) demonstrates a trend to lower the expression of these genes compared to VLD (Figure 6). L-Cs 10 LD treatment particularly alters *MnSOD* significantly (Figure 6, 2.34-fold decrease, *p* < 0.05) when compared to VLD. This specific downregulation of *MnSOD* suggests a potential mechanism of action or cellular adaptation that warrants further investigation.

### 2.6. L-Cycloserine Modulates Expression of Specific Proteins in Light-Damaged BALB/c Mice Retina

**Figure 7:**
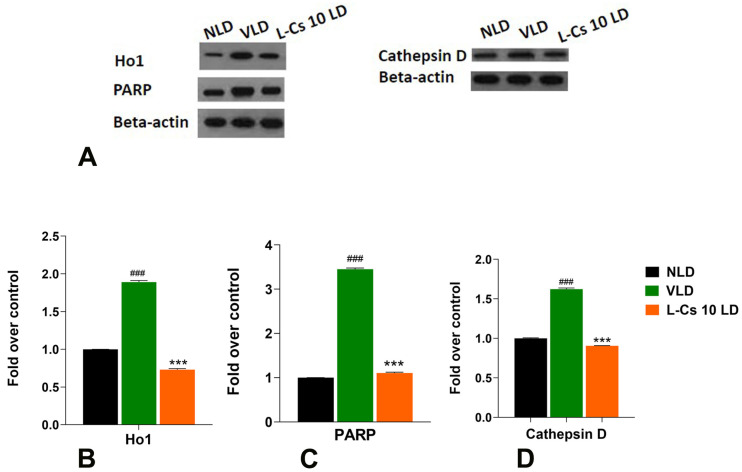
L-Cycloserine modulates expression of retinal proteins in light-damaged BALB/c mice. (**A**): Expression and quantification of heme oxygenase 1 (Ho1), Poly (ADP-ribose) polymerase (PARP) and Cathepsin D in BALB/c retina from no light-damaged control (NLD), vehicle-injected light-damaged (VLD) and L-Cycloserine (10 mg/kg) treated light-damaged (L-Cs 10 LD) group, 0 h after light damage. Quantification of (**B**): Ho1 (**C**) PARP (**D**): Cathepsin D in retinal tissue obtained with densitometric analysis and normalized with β-actin. (n = 3; # represents significance between NLD and VLD; ### *p* < 0.001, by student *t*-test; * represents significance between VLD and L-Cs 10 LD; *** *p* < 0.001, by student *t*-test).

The expression of specific proteins involved in cellular antioxidant and cell death pathways was also assessed immediately after light damage in all three groups of mice: control no light-damaged (NLD); vehicle-injected light-damaged (VLD) and L-Cycloserine-injected (L-Cs 10 LD) light-damaged group. Western blot analysis was performed to evaluate protein expression, and subsequent quantification was carried out through densitometric analysis (Figure 7). Light damage causes a significant upregulation in all three proteins investigated: heme oxygenase 1 (Ho1) (Figure 7B, 1.89-fold, *p* < 0.001), Poly(ADP-ribose) polymerase (PARP) (Figure 7C, 3.45-fold, *p* < 0.001), and Cathepsin D (Figure 7D, 1.62-fold, *p* < 0.001). However, treatment with L-Cycloserine (L-Cs 10 LD) causes the expression of all three proteins to be significantly reduced (*p* < 0.001, Figure 7B, C, and D, respectively).

## 3. Discussion

This study investigates the effect of inhibiting ceramide biosynthesis using a previously unexplored small molecule inhibitor L-Cycloserine, on light-induced photoreceptor cell death in BALB/c mice. The association between ceramide accumulation and retinal cell death has been highlighted in several reports [7,8,9,19]. In our earlier investigation, we explored the effect of L-Cycloserine on mouse photoreceptor-derived 661 W cells subjected to H_2_O_2_-induced oxidative stress [14]. The findings indicated that L-Cycloserine treatment significantly reduced bioactive ceramide and associated sphingolipids, showcasing effective inhibition of de novo ceramide biosynthesis and protecting against oxidative stress induced by H_2_O_2_ [14]. As part of our ongoing efforts to target ceramide biosynthetic pathways to mitigate ceramide accumulation and protect retinal photoreceptor cells from light-induced degeneration [7,13,19], we hypothesized that L-Cycloserine could inhibit ceramide biosynthesis, thereby protecting photoreceptor cells in BALB/c mice retina from light-induced degeneration.

Light-induced retinal degeneration (LIRD) mechanisms involve oxidative stress in the retina [31,32]. Excessive generation of ROS that is not regulated by the intrinsic defense system is attributed, at least in part, to the increased activity of the visual cycle during and after light exposure, leading to photoreceptor cell death through apoptosis [32]. Light exposure also induces inflammatory responses involving macrophages, cytokines, and eicosanoid mediators, which contribute to retinal pathology and conditions like AMD [28]. Furthermore, studies suggest that this inflammation due to photochemical retinal injury, which primarily affects the retinal pigment epithelium and photoreceptor layers, can be mitigated pharmacologically, such as through selective protection by dexamethasone [42]. Bioactive sphingolipids, including ceramide and its metabolites, contribute to retinal inflammation and degeneration by influencing cellular processes such as apoptosis, angiogenesis, and neovascularization [9]. This highlights their potential as therapeutic targets in retinal diseases; however, the precise mechanisms linking ceramide to inflammatory pathways have still not been fully explored and require further investigation.

The LIRD model used in this study progresses faster than degeneration in many animal models, making it a common choice for evaluating potential neuroprotective compounds [43]. This model allows for synchronized and regulated cell death, enabling the identification of biochemical markers associated with different phases of apoptosis [28]. The characteristic features of LIRD include the thinning of the ONL and the loss of PR cells, aligning with the pathology observed in retinal degeneration [19]. Previous studies have indicated an association between LIRD and the activation of the de novo pathway for ceramide production, which is the primary route leading to ceramide accumulation in light-stressed retinas [19,44]. 

The light damage protocol in our study involved IP administration of the drug 0.5 h before light damage, followed by ERG assessment 7 days post-light damage, to evaluate photoreceptor function [19]. By the 7th day, macrophages clear the dead photoreceptors, and the remaining nuclei in the ONL signify surviving photoreceptors [19]. The light damage condition, involving exposure to 1500 lux light intensity for 6 h, was established through a trial-and-error approach to achieve optimal damage feasible for mitigation by L-Cycloserine treatment. The IP dose of L-Cycloserine was chosen empirically based on previous mouse studies [45], and the results demonstrate significant damage induced by light (VLD group) and L-Cycloserine mediated protection from light-induced damage (L-Cs 10 LD group) as per Figure 1. 

The investigation for the optimal dose of L-Cycloserine providing maximum protection from light-induced damage employed the same light damage protocol, followed by ERG and histology at 7 days post-light damage, to assess photoreceptor function and morphology, respectively. The parabolic dosing effect observed in the ERG study with a higher dose of 40 mg/kg showing compromised protection might suggest the critical role of ceramide in cell membrane integrity and function [46]. Recent associations of ceramide deficiency with retinal dysfunction and degeneration further underscore the importance of maintaining ceramide homeostasis in the retina [47,48]. Consequently, the 10 mg/kg dose of L-Cycloserine appears to strike a balance to maintain this ceramide homeostasis, exhibiting maximum therapeutic effect in terms of protection from light-induced damage while minimizing toxic effects observed at higher doses like 40 mg/kg (Figure 2).

To further explore the reduced efficacy of L-Cycloserine at 40 mg/kg in preserving photoreceptor function and morphology in the presence of light, mice were exclusively injected with the most effective (10 mg/kg) and the least effective (40 mg/kg) doses of the drug without any light exposure. The control group of mice was injected with a vehicle (sterile water). Subsequently, ERG and histology were conducted after 7 days, following the established experimental timeline. The absence of a dose-dependent toxic effect of the drug in either case of photoreceptor function and morphology (Supplementary Figures S1 and S2, respectively) after seven days from administration suggests that the observed toxic effect in the presence of light may be a synergistic outcome of intense light exposure and ceramide inhibition occurring at a high dose of the ceramide biosynthesis inhibitor, L-Cycloserine. This points to the disruption of the natural homeostasis of ceramide, which is crucial for cell integrity, especially in a stressful condition like intense light exposure. It also might suggest the possibility of activating other signaling pathways involving ceramide biosynthesis, warranting further investigation.

Evaluating the biodistribution of the drug across various tissues and blood plasma helps in comprehending the pharmacokinetic behavior of L-Cycloserine (Figure 4). L-Cycloserine is a small molecule with a molecular weight of 102 Da, and our results suggest it can pass through the blood–brain barrier (BBB), consistent with previous findings [49]. The anticipated low physiological retention of this drug by elimination from the body by around 3 h is attributed to the drug’s hydrophilic nature (log *p*-value of −0.9) [50], rendering it highly soluble in blood plasma and urine. Reports suggest that 50–70% of the drug is excreted unchanged directly via urine [51], aligning with the observed rapid elimination. Cellular uptake likely occurs through passive diffusion through the paracellular pathway due to its small size [50]. However, the irreversible inhibition of the SPT enzyme by L-Cycloserine [49] may contribute to a longer therapeutic effect, preserving retinal function for at least 7 days after a single administration, despite the observed short retention time of the free drug in the body (Figure 4).

Subsequent evaluation of L-Cycloserine-induced protection of BALB/c mice retinal photoreceptors from light-induced degeneration involved a detailed sphingolipid analysis immediately after light damage (LD + 0) and 6 h after light damage (LD + 6) (Figure 5). The 10 mg/kg dose of L-Cycloserine was chosen as it offered the maximum protection against light-induced degeneration in previous experiments. The results demonstrate an increase in the relative combined percentage of ceramide and MHC upon light exposure, which supports our hypothesis, indicating ceramide upregulation induced by light damage (Figure 5A,B,D,E). Excessive bioactive ceramide, beyond the normal homeostatic levels, undergoes conversion to non-bioactive MHC by adding sugar to the ceramide backbone [52]. Ceramides destined for conversion to MHCs usually reach the Golgi via vesicular transport, followed by the addition of sugar moieties to very long-chain ceramides, catalyzed by glycosyl-transferases at the cytosolic surface of the Golgi [52]. The results also demonstrate that the relative combined percentage of ceramide and MHC continues to increase with time, as seen in VLD 0 vs. VLD 6 data (Figure 5B,E), indicating ongoing induction of ceramide biosynthesis for up to 6 h in agreement with our previous study [19]. Time-dependent progressive sphingolipid analysis for 24 h after light damage in the future can further delineate how ceramide level changes with time in the BALB/c mice LIRD model. 

The significant increase in C16:0 DH ceramide species on light exposure (Figure 5H), which is an integral intermediate in the de novo ceramide biosynthesis, emphasizes the possibility of activation of the de novo ceramide biosynthesis pathway. This is consistent with our prior studies [11,19,53,54], further supporting the notion that light exposure induces de-novo ceramide biosynthesis [19,55]. Comparison between ceramide species immediately (0 h) and 6 h after light damage indicates a potential sustained activation of the de novo pathway, especially with significantly increased levels of C16:0 DH (*p* < 0.05, Figure 5H). Additionally, our extensive lipidomics data reveals that light-induced stress increases short-chain ceramide species, thereby decreasing long-chain ceramide species, a pattern often associated with inflammatory conditions [14,56]. This indicates an elicitation of an inflammatory response by light-mediated oxidative stress conditions [57].

Evaluation of antioxidant, apoptotic, and sphingolipid pathway genes (Figure 6) and selective proteins (Figure 7) immediately after light damage provides insights into the impact of light and L-Cycloserine treatment on these pathways. Light-induced stress conditions (VLD) significantly upregulate the expression of *Fosl* (*p* < 0.05), *Mt2* (*p* < 0.01), and *Lcn2* (*p* < 0.05), which are all associated with antioxidant and/or apoptotic pathways [58,59,60] (Figure 6). However, the precise mechanisms behind these actions and the exact role of ceramide in these processes require further experimentation. 

Light-induced stress conditions (VLD) significantly increase the expression of Ho1 in BALB/c retinal tissue (*p* < 0.001) immediately after light damage (Figure 7B). Ho1 serves as an intracellular antioxidant and represents the stress-induced isoform of heme oxygenases [61]. It rapidly catalyzes the breakdown of heme, generating carbon monoxide, free ferrous iron, and biliverdin [62]. Ho1 is upregulated in reaction to oxidative stress and serves as an endogenous factor in mitigating inflammatory damage caused by stress [63]. Moreover, under light-induced stress conditions (VLD), there is a significant induction of PARP expression (*p* < 0.001, Figure 7C). PARP is an enzyme activated in response to cell death and serves as a marker for the parthanatos-mediated cell death pathway [64]. PARP activation is linked with apoptosis-induced conditions such as cerebral ischemia, inflammation, and oxidative stress injuries [64]. PARP has also been implicated in ceramide-induced apoptosis, with previous studies reporting its involvement in photoreceptor cell death [12]. These results also align with our earlier findings related to the effect of H_2_O_2_-induced oxidative stress on these selected proteins and subsequent L-Cycloserine co-treatment in 661 W mouse-derived photoreceptor cells [14]. 

Furthermore, under light-induced stress conditions (VLD), there is a significant induction of Cathepsin D expression (*p* < 0.001, Figure 7D). Cathepsin D, a major intracellular aspartyl protease, plays a role in mediating apoptosis induced by interferon-gamma and TNF-alpha [65]. While caspase proteases are well-known effectors of apoptotic pathways, recent evidence suggests that non-caspases, including cathepsins, also contribute to cell death [65]. Translocation of cathepsins from lysosomal compartments to the cytosol early during apoptosis has been reported, indicating their involvement in cell death pathways. There is also evidence that initiation of apoptosis by non-oxidant compounds, like light, involves the early release of cathepsins B and D, resulting in activation of phospholipase A2 (PLA2) and increased production of mitochondrial oxidants [66,67,68]. Cathepsin D has also been linked to ceramide-induced pro-inflammatory and pro-apoptotic pathways, connecting ceramide involvement in diseases such as diabetes mellitus [69]. These biochemical findings thus support the hypothesis that light damage activates cellular antioxidative defense mechanisms by upregulating relevant genes and proteins in BALB/c mice retina, implicating induced oxidative stress upon light exposure. Importantly, this effect is mitigated in the presence of L-Cycloserine, highlighting its protective role against light-induced ceramide-mediated cell death.

## 4. Materials and Methods

### 4.1. Animal Care and L-Cycloserine Treatment

All animals used in this study were bred and raised following the University of Tennessee Health Science Center (UTHSC) vivarium guidelines for animal housing. BALB/c male and female mice were born and raised in a dim cyclic light environment (5–10 lux; 12 h dark/12 h light). For experiments, 2-month-old mice were exposed to damaging light (white cool light) for 6 h (6 PM to 12 AM) at an intensity of 1500 lux. L-Cycloserine (Cat # C-1159; Millipore Sigma, St. Louis, MO, USA) dissolved in sterile water was administered intraperitoneal (IP) injection at 10 mg/kg, 0.5 h before light exposure. A dose–response study was also conducted to determine the most protective dose, utilizing 5, 10, 20, and 40 mg/kg of L-Cycloserine following the same protocol. Littermate mice injected with sterile water were the negative control, while healthy mice not exposed to damaging light were used as the positive control. Retinal functions were assessed seven days post-light damage using ERG. Subsequently, after ERG, mice were euthanized, and retinas were harvested for histopathological analyses. All procedures were performed according to the Association for Research in Vision and Ophthalmology Statement for the Use of Animals in Ophthalmic and Vision Research and thoroughly reviewed and approved by the UTHSC Institutional Animal Care and Use Committee (IACUC). 

### 4.2. Electroretinography (ERG)

Following light exposure, mice were placed back into dim cyclic light conditions (5–10 lux) for a 7-day recovery before ERG recordings were conducted. Both scotopic and photopic flash ERGs were recorded using the Celaris ERG system (Diagnosys LLC, Lowell, MA, USA), as previously outlined [70,71]. Mice underwent overnight dark adaptation and were prepared for ERG under dim red light. Anesthesia was induced via IP injection of ketamine (100 mg/kg; Covetrus, Chicago, IL, USA) and xylazine (5 mg/kg; Covetrus, Chicago, IL, USA) based on body weight. To dilate the pupil, a combination of 1% (*w*/*v*) Atropine (Amneal Pharmaceuticals, Bridgewater, NJ, USA) and 1% (*w*/*v*) Tropicamide (Akorn Inc., Lake Forest, IL, USA), along with 0.5% (*w*/*v*) Proparacaine HCl (Alkon Lab, Fort Worth, TX, USA) as local anesthetics, was applied to the cornea. A heating pad set at 37 °C was used throughout the procedure to maintain the mice’s warmth and body temperature.

For ERG measurement, designated electrodes were placed on each cornea, and the ERG was conducted using the TOUCH/TOUCH protocol developed by the manufacturer. Scotopic ERGs were conducted with four flash stimuli at intensities of 0.01, 0.1, 1, and 10 cd.s/m^2^. The a-wave amplitude was measured from the pre-stimulus baseline to the a-wave trough, while the b-wave amplitude was measured from the a-wave’s trough to the b-wave’s peak. For photopic ERG, mice underwent light adaptation (3 min at 10 cd.s/m^2^), followed by two flash stimuli at intensities of 3 and 10 cd.s/m^2^, and a 10 Hz flicker response.

To assess the impact of L-Cycloserine on retinal function, healthy 2-month-old BALB/c mice were also injected (IP) with L-Cycloserine at doses of 10 and 40 mg/kg, without exposure to light. Subsequently, they were returned to their native dim cyclic environment and subjected to ERG after 7 days using the same protocol as described above.

### 4.3. Histology 

After the completion of ERG recordings, euthanasia was administered to all mice using carbon dioxide asphyxiation following previously published methods [30,72]. In brief, eyes were harvested, marked with a tattoo dye at the 12 o’clock position on the limbus, and then fixed. Five-micron-thick sections were made along the vertical meridian through the optic nerve head (ONH). The number of outer nuclear layer (ONL) nuclei was counted at 0.24 mm distances from the center of the ONH to the inferior and superior ora serrata on hematoxylin and eosin-stained (H&E) sections, and the results were presented as a spider diagram.

### 4.4. Bio Distribution Study of L-Cycloserine in BALB/c Mice

Two-month-old BALB/c mice were divided into four different groups for the pharmacokinetic study. L-Cycloserine, dissolved in sterile water, was administered by IP route at a dose of 20 mg/kg. Each group of mice corresponded to a time point: 1, 3, 6, and 12 h after L-Cycloserine dosing. From each mouse, 0.5 mL blood samples were collected by cardiac puncture in a tube containing 20 µL of 0.5 M EDTA solution. The collected samples were then placed on ice for 4 h and centrifuged at 2500 rpm for 25 min at 4 °C. The resulting supernatant was collected as plasma and stored at −80 °C until further analysis. Tissues (brain, liver, and retina) were also harvested from each mouse, rinsed with 0.9% physiological saline solution to remove the blood, blotted dry with filter paper, accurately weighed, and subsequently stored at −80 °C until further analysis.

Plasma protein precipitation method was used for the sample pretreatment. A 50 μL amount of plasma samples was spiked with 200 μL of methanol containing 100 ng/mL of the internal standard (IS), caffeine. The mixture was vortexed and then cradled for 30 min at 4 °C followed by centrifugation at 10,000× *g* at 4 °C for 10 min. The supernatant was collected, and 10 µL was injected into the LC-MS/MS system for analysis. Each weighed tissue sample was thawed and homogenized in PBS at a 1:4 ratio (*w*/*v*). Subsequently, 25 μL of the homogenized sample combined with 200 µL of methanol containing 100 ng/mL of IS was vortexed and then cradled for 30 min at 4 °C. The mixture was then further processed using the same procedure as the plasma samples. A known drug concentration was used to spike plasma or homogenized tissue from control BALB/c mice with no treatment to prepare the standard curve. The subsequent steps followed the same procedure described above for the LC-MS/MS system analysis.

### 4.5. L-Cycloserine Quantification by LC-MS/MS

LC-MS/MS analysis was carried out using Sciex (Framingham, MA, USA) 5500 TripleQuad Mass Spectrometer coupled with Shimadzu (Columbia, MD, USA) LC20ADXR binary pumps, Shimadzu SIL20ACXR autosampler and Shimadzu CTO20AC column oven. A Supelco Ascentis Express C18, 2.7 mm, 50 × 4.6 mm HPLC column was used. The mobile phase of pure water (A) and acetonitrile (B) was eluted at a combined flow rate of 0.6 mL/min. The HPLC time program started at 15% of mobile phase B from 0 to 1.5 min, increased from 15% to 95% of mobile phase B from 1.5 to 1.6 min, and kept at 95% of mobile phase B from 1.6 to 2.1 min, and then decreased from 95% to 15% of mobile phase B from 2.1 to 2.2 min. The time program stopped at 2.5 min. The sample output from the column between 0.5 and 1.5 min was diverted to the ion source of the Mass Spectrometer. Before 0.5 min and after 1.5 min, the output was diverted to a waste bottle. There was an equilibration time of 1.5 min at the initial condition before each run.

Multiple Reaction Monitor (MRM) at positive mode was used for the MS/MS detection. The Ionization Spray capillary voltage was at 5.5 kV; the ion source temperature was at 500 °C; and CUR, CAD, GS1, and GS2 settings were at 20.0, 8.0, 50.0, and 50.0 PSI, respectively. Caffeine was used as IS in the LC-MS/MS measurement for L-Cycloserine. Compound-specific parameters are listed in Table 1.

### 4.6. Mass Spectrometry Analysis of Sphingolipids

For sphingolipid analysis, retinas were harvested from each animal group: control with no light damage (NLD), vehicle-treated light damaged (VLD), and L-Cycloserine 10 mg/kg treated light damaged (L-Cs 10 LD) group at 0 h and 6 h after light exposure. The harvested retinas were then stored at −80 °C and later sent for analysis to the lipidomic core facility at the University of Virginia, Charlottesville, VA, USA. Sphingolipids in retinal tissue were quantified and analyzed following previously published procedures [7,70,73,74,75]. Sphingolipid separation involved the utilization of UPLC ESI-MS/MS and was detected through a targeted assay using an AB Sciex Triple Quad 5500 Mass Spectrometer. Using positive ion mode and multiple-reaction monitoring, distinct species of sphingolipids were identified based on their retention time and *m*/*z* ratio. The semi-quantitative determination of each species was based on peak area measurements of internal standards spiked in the samples. Resulting values were reported in total picomoles and as mole percentages to standardize the data for comparison [7,70,73,74,75].

### 4.7. Gene Expression Analysis by Quantitative RT-PCR 

Following the manufacturer’s protocol, RNA was isolated and purified from frozen mice retinal tissues using the PureLink TM Micro-to Midi Total RNA Purification System from Invitrogen (Thermo Fisher Scientific, Carlsbad, CA, USA). Equal quantities (1.0 μg) of total RNA from each tissue were converted to first-strand cDNA using Superscript IV First-Strand Synthesis System (Invitrogen-Thermo Fisher Scientific, Vilnius, Lithuania) for reverse transcriptase polymerase chain reaction (RT–PCR). First-strand cDNA was used for quantitative RT-PCR (qRT-PCR). Primers for qRTPCR were designed in such a way that they spanned at least one intron, which eliminated the chance of amplification from residual genomic DNA contamination. The primer sequences are provided in Table 2. Quantitative PCR and melt curve analyses were performed using PowerUp SYBR Green Master mix from Thermo Fisher Scientific (Vilnius, Lithuania) and an iCycler machine (QuantStudio 3 from Thermo Fisher Scientific). The relative quantities of the expression of the genes of interest in different samples were calculated with the comparative Cq (threshold cycle) value method [76], with normalization against the housekeeping genes, Glyceraldehyde 3-phosphate dehydrogenase (*Gapdh*) and Ribosomal Protein L19 (*Rpl19*).

### 4.8. Western Blot Analysis

Total retinal proteins were isolated from mice retina by sonicating in T-PER reagent (Pierce, Rockford, IL, USA) containing a protease inhibitor cocktail (Roche, Indianapolis, IN, USA) and then centrifuging at 10,000× *g* for 15 min at 4 °C to collect the supernatants. After the protein concentrations were determined using BCA reagent (Pierce, IL, USA), equal aliquots (30 μg) of protein samples were applied to 10% sodium dodecyl sulfate-polyacrylamide gels (Invitrogen, Carlsbad, CA, USA) and electrophoretically separated. Resolved proteins were electrophoretically transferred to nitrocellulose membranes (Bio-Rad, Hercules, CA, USA) and blocked with 5% bovine serum albumin (BSA) for 2 h at room temperature. The membranes were incubated with anti-heme oxygenase 1 (Ho1) (1:1000; Cell Signalling, Danvers, MA, USA), anti-PARP (1:1000; Cell Signalling, Danvers, MA, USA), anti-Cathepsin D (1:100, Santa Cruz Biotechnology, CA, USA) and anti-β-actin (1:1000; Cell Signalling, Danvers, MA, USA) antibodies for 16 h at 4 °C, after which they were incubated with the appropriate peroxidase-linked secondary antibody for 1 h at room temperature. Chemiluminescence signals were detected by ECL detection reagent (Pierce; Thermo Scientific, Rockford, IL, USA) and imaged using Odyssey Imaging system (LI-COR). Densitometric analysis was performed using the manufacturer’s analysis software and was normalized to β-actin.

### 4.9. Statistical Analyses

Statistical analyses were performed using GraphPad Prism 8 (GraphPad Software, San Diego, CA, USA). The quantitative data are expressed as mean ± standard error of the mean (SEM) for each group. One-way or two-way ANOVA, Student’s *t*-tests (with Welch’s correction), and paired *t*-tests were performed to assess the differences between means.

## 5. Conclusions

In summary, this study demonstrated that L-Cycloserine, previously identified as a serine palmitoyl transferase (SPT) inhibitor [10,17,38,40], effectively protected the photoreceptor cells in BALB/c mice from light-induced retinal degeneration. Our findings suggest that targeting ceramide synthesis may be a promising approach for treating retinal degenerative diseases. However, further comparative studies on the inhibition profiles of L-Cycloserine across various pyridoxal 5′-phosphate (PLP)-dependent enzymes, such as transaminases, racemases, and decarboxylases, would provide valuable insights and clarify its specificity concerning ceramide synthesis inhibition. It is also important to note that the molecular pathology assessed by whole retinal analysis may not fully align with ERG or histology data, which reflect outcomes specifically in photoreceptor cells. Additionally, a more comprehensive time series lipidomics study is required to precisely determine the time points at which ceramide levels are reduced. Further investigations in genetic models or higher animals are warranted to expand on L-Cycloserine’s therapeutic potential in ceramide-induced degenerative diseases. This study nevertheless provides a foundation for novel therapeutic strategies targeting bioactive ceramides in retinal degeneration.

## Figures and Tables

**Table 1:** Compound-specific LC-MS/MS parameters.

Compound	DP (eV)	CE (eV)	Q1 Mass	Q3 Mass	T_R_ (min)
L-Cycloserine	6	11	103.1	75	0.69

DP: Decluster Potential; CE: Collision Energy; Q1: Molecular Ion Mass; Q3: Product Ion Mass; T_R_: Retention Time.

**Table 2:** Sequence of the primers used for RT–PCR.

Gene	Forward Primer (5′-3′)	Reverse Primer (5′-3′)
*Trx1*	GTGTGGACCTTGCAAAATGA	CCCAACCTTTTGACCCTTTT
*Cfos*	GAAACGGAGAATCCGAAGG	TGGGCTGCCAAAATAAACTC
*Fosl*	AGAGCGGAACAAGCTAGCAG	CAAGTACGGGTCCTGGAGAA
*MnSOD*	CTGGACAAACCTGAGCCCTA	CTGTAAGCGACCTTGCTCCT
*Mt2*	GCCTGCAAATGCAAACAAT	CGGAAGCCTCTTTGCAGAT
*Lcn2*	CCAGTTCGCCATGGTATTTT	GCTCTCTGGCAACAGGAAAG
*Spt2*	CATTGAGTCCAGAGCCAGAT	ACACACTGTCCTGGGAGGAA
*Sphk1*	GATGCATGAGGTGGTGAATG	AACAGCAGTGTGCAGTTGAT

## Data Availability

Data are contained within the article and Supplementary Materials.

## Supplementary Materials

The following supporting information can be downloaded at: https://www.mdpi.com/article/10.3390/ijms252413389/s1.
