# Supplementary material for: Inhibiting De Novo Biosynthesis of Ceramide by L-Cycloserine Can Prevent Light-Induced Retinal Degeneration in Albino BALB/c Mice
Source: Int J Mol Sci. 2024 Dec 13;25(24):13389. doi: 10.3390/ijms252413389 (PMC11676690; doi:10.3390/ijms252413389)
Supplement: Supplementary file 1 [file ijms-25-13389-s001.zip › ijms-3228013-supplementary.pdf]

Article

Inhibiting de novo biosynthesis of ceramide by L-Cycloserine can prevent light-induced retinal degeneration in albino BALB/c mice

Faiza Tahia[1,2], Dejian Ma[1], Daniel J. Stephenson[3], Sandip K. Basu[2], Nobel A. Del Mar[2], Nataliya Lenchik[2], Harry Kochat[4], Kennard Brown[4], , Charles E. Chalfant[3,5], Nawajes Mandal[1,2,6,7,*]

[1] Department of Pharmaceutical Sciences, University of Tennessee Health Science Center, Memphis, TN, 38163, USA
[2] Department of Ophthalmology, University of Tennessee Health Science Center, Memphis, TN, 38163, USA
[3] Departments of Medicine and Cell Biology, University of Virginia School of Medicine, Charlottesville, VA, 22903, USA
[4] Plough Center for Sterile Drug Delivery Solutions, University of Tennessee Health Science Center, Memphis, TN, 38163, USA
[5] Research Service, Richmond Veterans Administration Medical Center, Richmond VA, 23298, USA
[6] Department of Anatomy and Neurobiology, University of Tennessee Health Science Center, Memphis, TN, 38163, USA
[7] Memphis VA Medical Center, Memphis, TN, 38104, USA
* Corresponding author: Email: nmandal@uthsc.edu; Phone: +1-901-448-7740

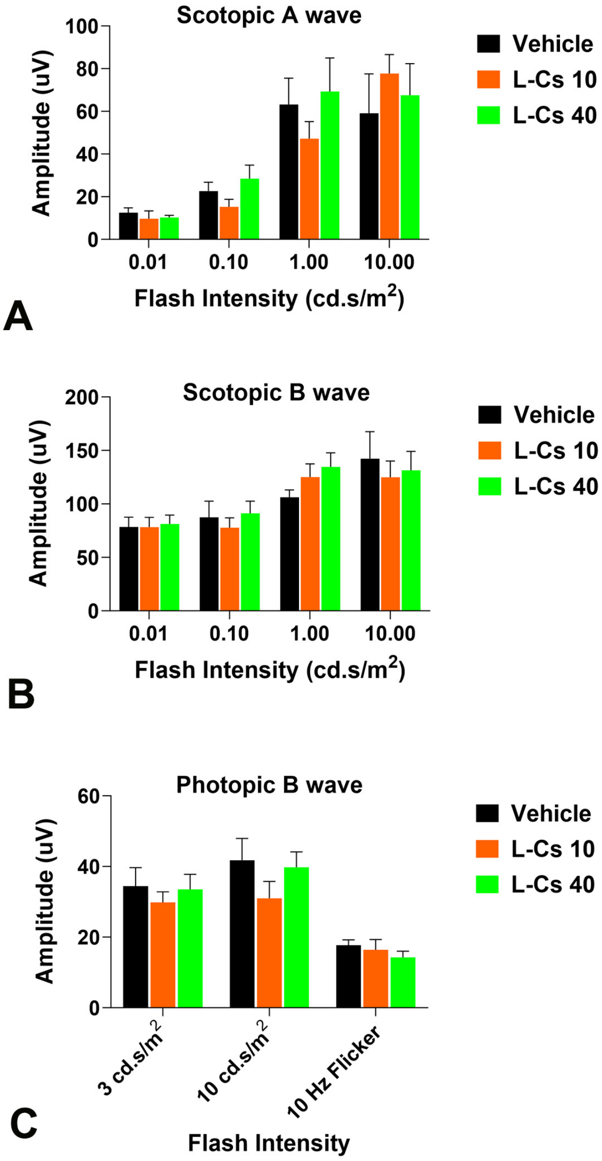

Figure S1: L-Cycloserine up to a dose of 40 mg/kg shows no toxicity in BALB/c mice retina. ERG analysis shows Scotopic a-wave (A), b-wave (B) and Photopic b-wave (C) from Vehicle: vehicle (sterile water) injected group; L-Cs 10 and 40: L-Cycloserine-treated (10 and 40 mg/kg, respectively) group. (n = 5/group; values represent mean ± SEM; student t-test).

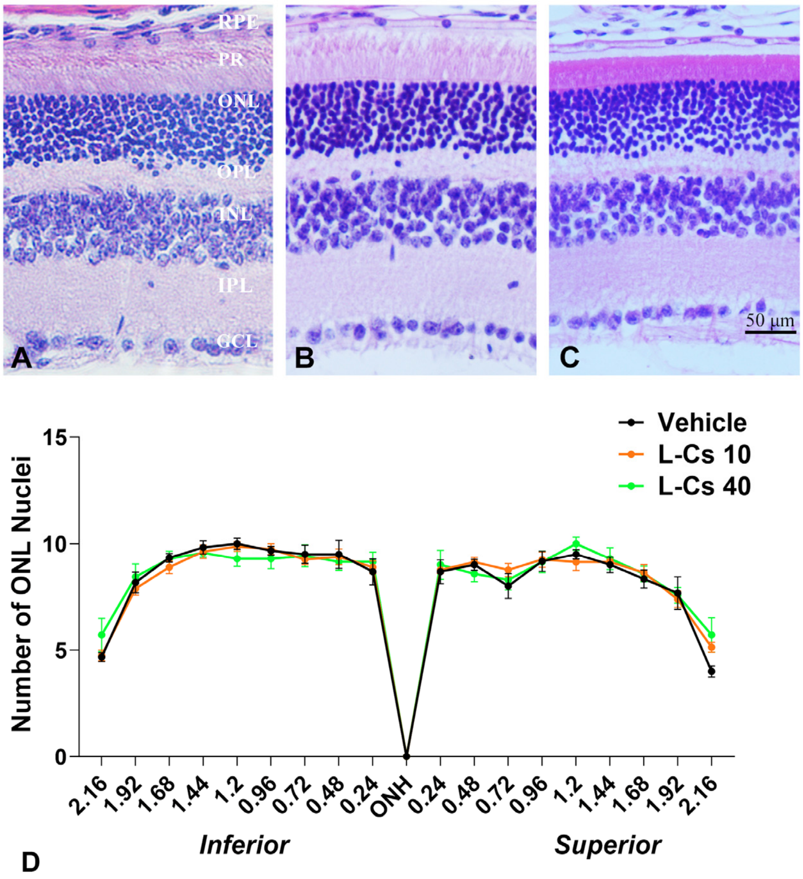

Figure S2: L-Cycloserine up to a dose of 40 mg/kg shows no toxicity in BALB/c mice retina. Representative retinal histological sections from each treatment: A (Vehicle): vehicle (sterile water) injected group; B (L-Cs 10): L-Cycloserine-treated (10 mg/kg) group; C (L-Cs 40): L-Cycloserine-treated (40 mg/kg) group. D: Quantitative morphometric measurement of ONL nuclei count from H and E-stained slides (n = 6 for Vehicle, 8 for L-Cs 10 and 7 for L-Cs 40). Values represent mean ± SEM; student t-test.
